# Prophylactic efficacy and safety of valproate versus topiramate for migraine: a systematic review

**DOI:** 10.1186/s12883-026-04990-7

**Published:** 2026-05-20

**Authors:** Saad Arsalan Wasti, Areesha Wasti, Muhammad Ali Abid, Muhammad Shamikh Shahid, Vicky Fu Hsuan Kuo, Sandra Elena Cano-Reyes, Muhammad Usman Iqbal, Andrii Vasylyshyn, Abouch Valenty Krymchantowski

**Affiliations:** 1 King Edward Medical University, Lahore, Pakistan; 2 Continental Medical College, Lahore, Pakistan; 3 National Taiwan University, Taipei City, Taiwan; 4 Bogotá D.C, Universidad Nacional de Colombia, Bogotá, Colombia; 5 Ivano-Frankivsk National Medical University, Ivano-Frankivsk, Ukraine; 6 Brazilian Academy of Headache and Orofacial Pain, Rio de Janeiro, Brazil; 7 Headache Center of Rio, Rio de Janeiro, Brazil

**Keywords:** Valproate, Topiramate, Migraine, Review

## Abstract

**Background:**

Migraine prophylaxis options commonly include valproate and topiramate. Although several randomised trials have directly compared these agents, their relative efficacy and safety remain underexplored. Existing reviews have largely focused on placebo-controlled or indirect comparisons and have not comprehensively synthesised the available head-to-head evidence. This systematic review, therefore, evaluates the comparative efficacy and safety of valproate versus topiramate using a comprehensive, multilingual search

**Methods:**

This review has been registered on the Open Science Framework (DOI: https://doi.org/10.17605/OSF.IO/ZC3S6). Databases and registers were searched to 9 September 2025. We included randomised controlled trials (RCTs) comparing valproate with topiramate in adult migraineurs. Due to the high risk of bias, evaluated at the outcome level using the revised Cochrane Risk of Bias 2 tool, a descriptive approach was adopted.

**Results:**

Eight trials were included (1659 participants; 72.2% female; mean ages 29.2–42.1 years). Two studies were in Chinese, one in Persian, and the rest were in English. Both drugs appeared to reduce migraine frequency meaningfully. Reductions in migraine duration, pain intensity, and disability measures (HIT-6, MIDAS) improved similarly with both drugs. The evidence base was insufficient to support a definitive efficacy advantage for either drug. Adverse events were common but mostly mild. Valproate use was often associated with increased appetite, drowsiness, and tremor, while topiramate use was commonly associated with paraesthesia and appetite loss.

**Conclusions:**

The head-to-head randomized evidence appears to suggest that both valproate and topiramate may be effective for migraine prophylaxis. However, the current evidence is insufficient to support definitive claims of superiority for either drug. Differences in adverse- event profiles may therefore play a key role in guiding individualised treatment decisions. High- quality, well-powered trials are needed.

**Supplementary Information:**

The online version contains supplementary material available at https://doi.org/10.1186/s12883-026-04990-7.

## Strengths and limitations of this study

- A comprehensive, multilingual search strategy was employed across multiple bibliographic databases, trial registries, websites and regional article indexes, reducing the risk of missing studies published in languages other than English or not indexed on major databases.
- The review focused exclusively on head-to-head randomised controlled trials, allowing direct comparison of valproate and topiramate without reliance on indirect or network-based evidence.
- Risk of bias was assessed at the outcome level using the Cochrane Risk of Bias 2 tool according to Cochrane recommendations, enhancing transparency in the appraisal of evidence quality.
- A meta-analysis was deliberately not performed due to substantial clinical and methodological heterogeneity and high risk of bias, in line with contemporary methodologic guidance, but this limited quantitative synthesis of effects.
- The review was restricted to aggregate published data, as individual participant data were unavailable, limiting the ability to adjust for baseline imbalances or explore subgroup effects.

## Background

Migraine is a highly prevalent neurologic disorder characterised by recurrent headaches, and is often accompanied by symptoms such as nausea and photophobia [1]. Globally, it affects more than a billion people, and has a substantial burden on the quality of life and healthcare systems worldwide [2]. Migraine prophylaxis aims to reduce the frequency, severity, and duration of migraine attacks while improving patient functioning. Amongst the various pharmacologic options, valproate and topiramate (both antiseizure medications) are widely used for migraine prevention, as supported by many clinical recommendations and guidelines [3–5]. Valproate has demonstrated efficacy in decreasing migraine frequency and severity in randomised controlled trials [6, 7]. Similarly, topiramate has been extensively studied and shown to significantly reduce migraine days and have higher responder rates compared to placebo in migraineurs [8]. Both drugs possess distinct mechanisms of action and side effect profiles, which are factors that may influence their clinical use and tolerability.

Several head-to-head randomised controlled trials (RCTs) have directly investigated valproate versus topiramate in migraine prophylaxis. However, their findings are inconsistent. For example, Zhang found topiramate to be significantly better than valproate in reducing migraine frequency between the two drugs [9]. Choudhary et al. [10], concluded that valproate yielded significantly better reductions in migraine frequency [10]. Other trials, such as that conducted by Bavrasad et al., 2010, found no significant differences between the two agents in reducing migraine frequency [11]. These mixed findings call for a comprehensive review of available trials to better understand the relative performances of the two drugs. Despite this, comparative reviews exploring their relative efficacy and safety remain limited [12–14]. Some network meta-analyses also exist, but are constrained by methodologic limitations such as insensitive search strings, language restrictions or incomplete database coverage which have led to exclusion of much of the available evidence base [15–17]. This has resulted in the inclusion of only one or two of the available trials in prior reviews, and, consequently, the conclusions are largely based on indirect evidence. Accordingly, a systematic review specifically focused on comprehensively identifying and synthesising head-to-head RCTs comparing valproate and topiramate, using sensitive and multilingual search strategies, is needed. Such a review may offer clearer insights into their comparative prophylactic efficacy and safety profiles and better support informed clinical decision-making.

In this context, the present systematic review aimed to evaluate the prophylactic efficacy and safety of valproate versus topiramate for migraine prophylaxis, incorporating a sensitive and comprehensive literature search across multiple databases and languages to capture relevant RCT evidence. This review sought to address limitations of previous syntheses by including non-English-language studies and by employing rigorous screening procedures to mitigate bias associated with omitted trials. The findings aim to inform clinical decision-making and identify priorities for future research in migraine prophylaxis.

## Methods

This systematic review was conducted in line with the guidelines outlined in the Cochrane Handbook for Systematic Reviews of Interventions [18], and reported according to the Preferred Reporting Items for Systematic Reviews and Meta-Analyses (PRISMA) statement of 2020 [19]. The completed PRISMA checklist is available in Supplementary Table S1. We registered this review on the Open Science Framework (OSF) (Registration DOI: https://doi.org/10.17605/OSF.IO/ZC3S6) [20]. We did not require ethical approval to conduct this review, as publicly available, previously published data were utilised.

### Data sources and search strategy

We searched PubMed, Embase, Scopus, Web of Science, Cochrane, ClinicalTrials.gov, World Health Organisation International Clinical Trials Registry Platform (WHO ICTRP) from inception to September 9, 2025. Additional studies were identified through citation chaining and searching websites. The detailed search strategies for each source are provided in Supplementary Table S2. The search string consisted of free-text keywords and controlled vocabulary terms related to valproate, topiramate and migraine. Literature searches were conducted in English, and supplemented through additional searches in Chinese, Persian, and Spanish. Relevant non-English articles were translated in consultation with native speakers having medical backgrounds.

### Study selection process

We imported all retrieved records into Rayyan software for screening [21]. After de-duplication, two authors independently screened the records first against titles and abstracts, and then against full texts. Literature screening followed a pre-specified set of inclusion and exclusion criteria. Conflicts arising during the screening process were resolved in consultation with a third author.

### Eligibility criteria

Inclusion criteria were: (1) Adult patients having migraine; (2) Usage of valproate as an intervention; (3) Comparison with topiramate as control; (4) Reporting relevant efficacy and/or safety outcomes; (5) Randomised controlled trials (RCTs) by design; (6) Any language.

Exclusion criteria were: (1) Studies enrolling animals, children, or patients not having migraine; (2) Not using valproate as an intervention; (3) Not comparing to topiramate; (4) Not reporting relevant efficacy and/or safety outcomes; (5) Other study designs such as case reports, reviews, quasi- studies, and case series.

### Data extraction

Two reviewers independently extracted data using pre-piloted Excel sheets. Discrepancies were resolved through discussion or through arbitration with a third reviewer if needed. Extracted data included study characteristics, participant demographics, drug characteristics, and outcome data.

### Risk of bias assessment

Risk of bias for each outcome was independently assessed by two reviewers using the revised Cochrane Risk of Bias 2 (RoB 2) tool [22]. The RoB 2 tool assesses risk of bias across five domains: randomisation process; deviations from intended interventions; missing outcome data; measurement of the outcome; and selection of the reported result. Risk of bias was judged for seven key outcomes: migraine frequency, duration, pain intensity, Headache Impact Test (HIT-6) score, Migraine Disability Assessment (MIDAS) score, responder rate (≥ 50% reduction in migraine frequency), and adverse events. Disagreements were resolved through consensus or through a third reviewer.

### Data synthesis

A statistical synthesis of the outcomes was planned to be conducted in R (version 4.5.1) using a random-effects model. For continuous outcomes, mean differences (MD) with 95% confidence intervals (CIs) were to be calculated, and for binary outcomes, risk ratios (RR) with 95% CIs were to be calculated. However, in line with recommendations from contemporary guidelines and Chap. 12 of the Cochrane Handbook for Systematic Reviews of Interventions, we decided against the pre-specified meta-analysis that was mentioned in the protocol, and instead adopted a descriptive approach [18, 23–25]. The main reason for not conducting a meta-analysis was the extremely high risk of bias in the evidence base. This was compounded by substantial clinical, methodological, and statistical heterogeneity. A meta-analysis in this case would have been unreliable and could have led to spurious conclusions [24, 25].

### Evidence certainty

Certainty of evidence was appraised using the Grading of Recommendations Assessment, Development and Evaluation (GRADE) approach. This approach is a transparent and structured framework used to rate the certainty of evidence and the strength of healthcare recommendations. It assesses the quality of evidence for each patient-relevant outcome, starting with an initial rating of high for RCTs, which can be downgraded based on five domains: risk of bias, inconsistency, indirectness, imprecision, and publication bias. The final certainty is categorized into four levels: High, Moderate, Low, or Very Low. The findings were tabulated in a Summary of Findings (SoF) table.

## Results

### Study selection process

**Fig. 1:**
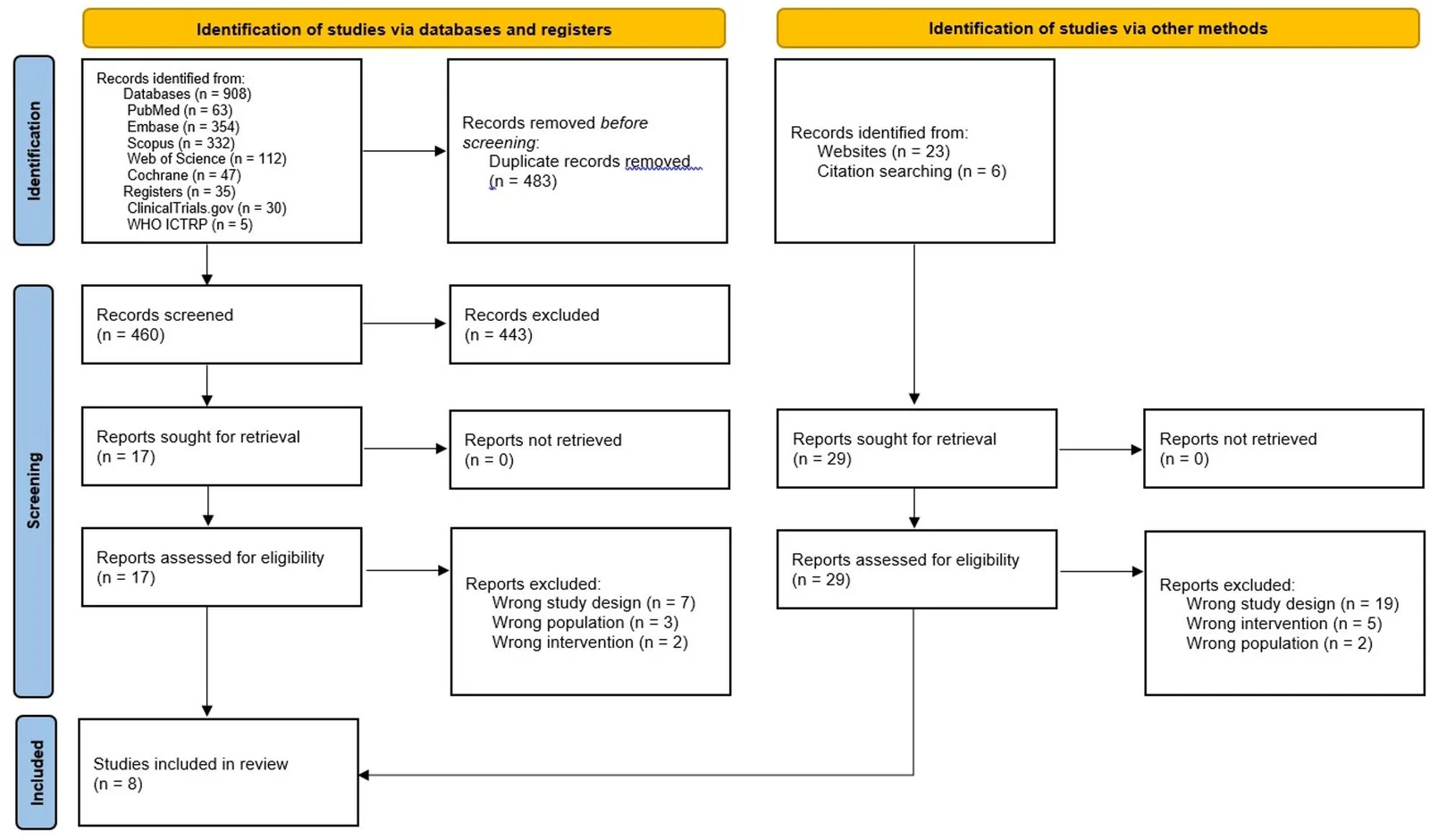
PRISMA flow diagram delineating the study selection process

A total of 908 records were identified through database searches, with an additional 35 records located through registers. After removing 483 duplicates, 460 unique records were screened by title and abstract, and 443 were excluded as they were not relevant to the present review (with the reasons including not studying the population of interest, not being of the eligible study design, and assessing interventions outside the scope of this review). The full texts of 17 articles from the database/register section were assessed for eligibility, and 5 were included. From other sources, a total of 29 records were obtained and 3 met our inclusion criteria. Ultimately, 8 studies were included in our review. The results of the study selection process are outlined in the Preferred Reporting Items for Systematic Reviews and Meta-Analyses (PRISMA) flow diagram, in (Fig. 1). 

### Characteristics of included studies

Tables 1 and 2 summarise the key characteristics of the included trials. Across the eight studies, publication years ranged from 2005 to 2025, and trials were conducted in Iran, Italy, Pakistan, China, Egypt, and the United Arab Emirates. Seven studies were single-centre, and one was multi-centre. Two studies were in Chinese, one in Persian, and the rest were in English. Follow-up durations varied widely, from 6 weeks to 5 months. In total, 1659 participants were included, with a female majority across the trials (72.2%), and mean ages varied from 29.2 ± 9.6 to 42.1 ± 8.9 years.

Valproate and topiramate dosing regimens varied greatly within and between studies. Durations of drug administration also showed noticeable differences (ranging from 1 month to 5 months). Migraine duration history was reported in some studies and spanned from 3.5 ± 2.2 to 8.2 ± 1.5 years, where reported. Overall, the included studies demonstrated considerable variation in diagnostic definitions, baseline characteristics, and dosing strategies.

**Table 1:** Table summarising key characteristics of the included studies

Study Characteristics												
Author	Year Of Publication	Country	Language	Center	Total Number Of Study Arms	Total Number Of Arms Of Interest	Sample Size			Migraine Characteristics	Diagnostic Criteria	Follow-up
							V	T	V + T			
Afshari et al. [26]	2012	Iran	English	Single	2	2	28	28	56	Valproate: MwA = 3, MwoA = 25 Topiramate: MwA = 6; MwoA = 22	International Headache Society (IHS)	Every 4 weeks (3 visits)
Bartolini et al. [27]	2005	Italy	English	Single	2	2	22	22	44	Episodic migraine without aura	International Headache Society (IHS)	1 month, 3 months
Bavrasad et al.[11]	2010	Iran	English	Single	2	2	35	35	70	1–6 migraine attacks per month for at least 1 year	International Headache Society (IHS)	Monthly, till 3 months
Choudhary et al.[10]	2017	Pakistan	English	Single	2	2	45	45	90	Not specified	Not specified	6 weeks
Fazlalizadeh et al. [28]	2009	Iran	Persian	Single	2	2	285	284	569	Unilateral headache in 309 patients, pulsating headache in 476 patients. Associated symptoms were common, with nausea and vomiting reported by 457 patients, photophobia by 486, and phonophobia by 472.	International Headache Society (IHS)	2 weeks, 1 month
Ma et al. [29]	2009	China	Chinese	Single	2	2	68	60	128	A definite history of migraine, with or without aura; disease duration of more than 6 months; at least 3 attacks per month; severe headache affecting work and daily life; attack intervals longer than 48 h.	International Headache Society (IHS)	5 months
Zeinhom et al.[30]	2025	Egypt, United Arab Emirates	English	Multi	2	2	300	300	600	Valproate: MwA = 86, Chronic Migraine = 51 Topiramate: MwA = 79, Chronic Migraine = 47	International Classification of Headache Disorders 3rd Edition (ICHD-3)	3 months (open-ended telephone conversations twice-weekly and face-to-face interviews in outpatient clinic once-monthly)
Zhang et al. [9]	2018	China	Chinese	Single	2	2	51	51	102	Chronic migraine	International Headache Society (IHS)	3 months

*Abbreviations*: *N/R* not reported, *MwA* migraine with aura, *MwoA *migraine without aura, *IHS *International Headache Society, *ICHD*-3 International Classification of Headache Disorders 3rd Edition, *BMI *body mass index, kg kilogram, kg/m^2^kilograms per square meter, mg/d milligrams per day, *SD *standard deviation, *V *valproate arm, *T *topiramate arm,* V + T *combined valproate and topiramate data (if applicable), *N *(%) number (percentage)

**Table 2:** Table summarising participant demographics and drug characteristics in the included studies

Author	Year Of Publication	Participant Demographics											Drug Characteristics			
		Age (Years)		Weight (kg)		BMI (kg/m^2^)		Females (*N*, %)		Dropouts From Study (%)	Mean Duration History of Migraine (Mean ± SD)		Valproate Group		Topiramate Group	
V		T	V	T	V	T	V	T	V		T	Dose	Duration	Dose	Duration	
Afshari et al. [26]	2012	29.2 ± 9.6	32.1 ± 10.2	62.3 ± 12.3	72.3 ± 15.4	N/R	N/R	22 (78.6%)	22 (78.6%)	26.30%	6.4 ± 8.1 Years	5.4 ± 5.1 Years	200 mg/d for the first week, then increased to 400 mg/d	12 Weeks	25 mg/d for the first week, then increased to 50 mg/d	12 Weeks
Bartolini et al. [27]	2005	41.9 ± 10.1	41.7 ± 9.8	N/R	N/R	N/R	N/R	18 (81.8%)	13 (59.1%)	10.20%	5.6 ± 1.4 Years	5.3 ± 1.5 Years	250 mg/d induction dose, 750 mg/d at end of titration phase (250 mg in morning/500 mg in evening)	3 Months	25 mg/d induction dose, 75 mg/day at end of titration phase (25 mg in morning/50 mg in evening)	3 Months
Bavrasad et al.[11]	2010	31.2 ± 5	30.1 ± 6	45–85		19–29		35 (100%)	35 (100%)	4.30%	N/R	N/R	400–600 mg/d	3 Months	50–75 mg/d	3 Months
Choudhary et al.[10]	2017	39.6 ± 9.3	42.1 ± 8.9	N/R	N/R	N/R	N/R	34 (75.6%)	31 (68.9%)	N/R	N/R	N/R	1000 mg/d in two divided doses	6 Weeks	100 mg/d in two divided doses	6 Weeks
Fazlalizadeh et al. [28]	2009	31.16 ± 8.8		N/R	N/R	N/R	N/R	430 (75.6%)		N/R	N/R	N/R	200 mg/d	1 Month	100 mg/d	1 Month
Ma et al. [29]	2009	35.6 ± 6.8	29.8 ± 13.5	N/R	N/R	N/R	N/R	22 (32.4%)	25 (41.7%)	N/R	4.8 ± 3.2 Years	5.5 ± 4.3 Years	Initial dose 125 mg/d, increased by 125 mg/d every 5 days until reaching a maintenance dose of 750 mg/d	5 Months	Initial dose of 50 mg/d, increased by 50 mg/d every 5 days until reaching a maintenance dose of 150 mg/d	5 Months
Zeinhom et al.[30]	2025	31 ± 9.7	33.3 ± 11.2	76.1 ± 12.4	76.3 ± 11.9	N/R	N/R	231 (77%)	222 (74%)	7.30%	3.5 ± 2.2 Years	3.5 ± 3 Years	Initial dose 500 mg once daily for 1 week, then twice daily from 8th day onwards	3 Months	Initial dose 50 mg once daily for 1 week, then twice daily from 8th day onwards	3 Months
Zhang et al. [9]	2018	38.4 ± 6.5	37.8 ± 7.2	N/R	N/R	N/R	N/R	25 (49%)	23 (45.1%)	N/R	8.2 ± 1.5 Years	7.9 ± 1.3 Years	Initial dose 400 mg/d, divided into two oral doses, with adjustments according to headache response; maximum dose ≤ 600 mg/d	3 Months	Initial dose 25 mg/d, divided into two oral doses; dose increased weekly by 25 mg, maximum ≤ 100 mg/d	3 Months

*Abbreviations*: *N/R* not reported, *MwA *migraine with aura, *MwoA *migraine without aura, *IHS *International Headache Society, *ICHD*-3 International Classification of Headache Disorders 3rd Edition, *BMI *body mass index, kg kilogram, kg/m^2^ kilograms per square meter, mg/d milligrams per day, *SD *standard deviation *V *valproate arm, *T *topiramate arm, *V + T* combined valproate and topiramate data (if applicable), *N *(%), number (percentage)

### Risk of bias assessment

We assessed the risk of bias for each outcome across five domains, and also rated an overall risk of bias judgement. The outcomes assessed were migraine frequency, responder rate, migraine duration, migraine pain intensity, migraine HIT-6 score, migraine MIDAS score, and adverse events. Across most outcomes, the overall risk of bias was judged as high. The risk of bias assessment per outcome is given in the supplementary file (Supplementary Figures SA1-SA14). Many studies provided insufficient detail on participant allocation. Blinding of participants, personnel, and outcome assessors was often inadequately reported or not done, introducing potential performance and detection bias. Given that the outcomes mostly relied on self-reported measures, this potentially introduces bias. Other important methodological limitations included reliance on per-protocol analyses and the lack of prospective trial registration in several studies.

## Outcomes

### Primary

#### Migraine frequency

Seven studies reported on the effects of the drugs on migraine frequency. Across all, both valproate and topiramate produced clinically meaningful reductions in attack frequency from baseline. Three studies [36, 11, 40] found similar improvements in both groups with no statistically significant between-group differences at any timepoint. Bartolini [27] observed a greater reduction with valproate at the 1-month assessment after reaching full-dose therapy, although this advantage disappeared by three months when both drugs showed large and comparable reductions. Two studies [10, 29] concluded that valproate was the more efficacious drug in this regard. In contrast, one study [9] reported a significantly larger reduction with topiramate after three months of therapy. Overall, although both drugs consistently reduced migraine frequency, the relative effectiveness differed across studies (Supplementary Tables S3 and S4).

### Responder rate

Seven studies reported responder rates for both groups. Across trials, responder rates in the valproate groups ranged from 22.3% to 95.5%, while responder rates in the topiramate groups ranged from 27.3% to 95.5%. In three trials, responder rates were broadly similar between groups. One study [11] reported no significant difference but did not provide numerical responder counts. One trial [30] showed slightly higher responder rates with topiramate compared with valproate (27.3% versus 22.3%), whereas two studies [39, 10] favoured valproate. Overall, the direction of effect was inconsistent across studies. The results of each study are given in Supplementary Table S5.

### Secondary

#### Migraine duration

Five studies reported on migraine duration, and all demonstrated meaningful within-group reductions over time. Two studies [11, 26]) reported substantial reductions in average attack duration in both arms, with no statistically significant difference between topiramate and valproate at any follow-up point. In contrast, [39] found that valproate produced a significantly greater reduction in attack duration over five months compared with topiramate. One study [30] also observed progressive shortening of attack duration in both groups, again without significant between-group differences. Only Zhang [9] reported a significant advantage for topiramate. Overall, both agents consistently reduced migraine duration, although individual studies differed regarding which drug achieved greater improvement (Supplementary Tables S3 and S4).

### Migraine pain score (migraine pain intensity)

Across the four trials that reported migraine pain intensity [9, 11, 30] all studies demonstrated significant within-group reductions in pain severity over the administration period in both topiramate and valproate arms. Three studies [9, 26, 30] reported significantly lower end-point Visual Analog Scale (VAS) scores with topiramate, indicating greater improvement in pain intensity. In contrast, Bavrasad [11] showed comparable Numeric Rating Scale (NRS) scores between groups (*P* = 0.259) despite significant within-group reductions. Overall, although available data suggest a pattern favouring topiramate for reducing migraine pain intensity, interpretation is restricted by heterogeneity and lack of baseline-adjusted results (Supplementary Tables S3 and S4). Evidence is insufficient to conclusively establish the superiority of topiramate in this regard.

### Migraine HIT-6 score

Across the two trials reporting effects on headache-related disability using the HIT-6 scale [26, 30] both topiramate and valproate produced clinically meaningful reductions from baseline. Afshari [26] observed substantial within-group improvements in both arms, with a larger reduction in the topiramate group (mean decrease from 64.5 ± 4.7 to 49.2 ± 8.1) compared with valproate (65.8 ± 5.0 to 57.2 ± 6.9; *P* = 0.00), although this outcome was not pre-specified in the trial registry, and was only reported at baseline and week 8 timepoints. Zeinhom [30] reported similar HIT-6 scores across groups at baseline, with modest, non-significant differences at 1 month, but significantly greater reductions favouring topiramate at both 2 months (*P* = 0.01) and 3 months (*P* = 0.004) (Supplementary Tables S3 and S4).

### Migraine MIDAS score

Across the three trials reporting MIDAS scores [11, 26, 27] all studies demonstrated substantial within-group reductions in migraine-related disability following administration with either topiramate or valproate. Afshari [26] reported a non-significant between-group difference (*P* = 0.128). It is important to note, however, that the outcome was not prespecified in the trial registry. Bartolini [27] similarly reported no significant intergroup difference. Bavrasad [11] found significant reductions in both groups by 12 weeks and noted a greater degree of improvement with topiramate, but did not provide post-intervention or change-from-baseline values and reported significantly imbalanced baseline scores. Overall, available evidence indicates that both topiramate and valproate likely substantially reduce migraine-related disability, but there is insufficient data to recommend one intervention over the other for improving migraine-related disability (Supplementary Tables S3 and S4).

### Adverse events

Adverse events were reported in all included studies except one, although the level of detail differed. Both valproate and topiramate were generally associated with mild to moderate side effects, with some variation in the type and frequency of events across trials. Valproate was most commonly linked to symptoms such as increased appetite, drowsiness, nausea, tremor, and hair loss. On the other hand, topiramate was more frequently associated with decreased appetite, paresthesia, and dizziness. Several studies reported discontinuations in both groups due to adverse effects. The overall incidence of adverse events by the two drugs was broadly comparable, though differences existed in the individual kinds of adverse events seen. The adverse events reported by each study are given in Supplementary Table S6.

### Evidence certainty

The certainty of evidence for all outcomes was assessed using principles from the GRADE framework. Overall, the certainty of evidence was judged to be very low across all key outcomes, including migraine frequency, responder rate, duration, pain intensity, disability measures, and adverse events (Supplementary Table S7). This was primarily due to serious concerns regarding risk of bias, including lack of blinding, per-protocol analyses, and incomplete outcome reporting. Additional downgrading was applied for inconsistency and for imprecision. For reference, very low certainty means that we have very little confidence in the effect estimate, and that the true effect is likely to be substantially different from the estimate of effect. As a result, confidence in the comparative effectiveness and safety of valproate versus topiramate remains limited, and the true effects could likely differ substantially from the observed findings.

## Discussion

This systematic review aimed to evaluate the efficacy of valproate and topiramate in migraine prophylaxis, focusing on migraine frequency, responder rates, migraine duration, pain intensity, and disability reduction. Eight studies were included, involving participants from diverse regions including Iran, Italy, Pakistan, China, Egypt, and the United Arab Emirates. Across the majority of studies, both drugs demonstrated substantial efficacy. However, the collective evidence remained insufficient to clearly establish the superiority of either treatment, as individual trials reported mixed findings. Some favoured valproate, others topiramate, and several showed no statistically significant differences. The variations observed in the included studies may be attributed to dissimilarities in study methodologies, dosing regimens, baseline migraine characteristics, and follow-up durations. Notably, the lack of change-from-baseline data in the trials hinders direct comparisons across studies, as noticeable baseline imbalances were present. It is important to note that topiramate, compared to placebo, has been shown to significantly reduce monthly migraine days [8, 31]. The same evidence is found for valproate providing a 50% or more reduction in monthly migraine days when compared to placebo [15, 32].

Responder rates varied widely for valproate and for topiramate. This inconsistency in response is likely due to the heterogeneity of the included trials, particularly regarding baseline migraine severity, differences in drug administration protocols, and the duration of administration. Despite this variability, the responder rates from the majority of studies suggest that both drugs are generally effective in providing relief to migraineurs.

Cui et al. have similarly shown that there is no significant difference between the responder rates for valproate and topiramate, although the responder rate in the valproate group was lower than that of topiramate [33]. The reduction in migraine duration was consistently observed across five studies, although the magnitude of improvement varied. Topiramate showed a marginal edge over valproate in some studies, particularly in terms of reducing pain intensity. However, the differences in pain intensity were not always statistically significant, indicating that both drugs are similarly effective in managing pain for most patients. Both valproate and topiramate contributed to meaningful improvements in disability scores, as measured by MIDAS and HIT-6. While both medications led to substantial reductions in disability, the lack of baseline-adjusted data and imbalanced baseline scores in some studies prevent definitive conclusions regarding the superior drug for improving disability outcomes.

Studies reported varied adverse effects from therapy with valproate and topiramate. These were consistent with the adverse events reported in the literature. Notably, in the largest included trial [30], a significantly higher proportion of participants in the valproate group experienced adverse events compared to the topiramate group (43% versus 22%). It should be noted, however, that significant differences in adverse event profiles were not consistently seen across all trials, and that whilst adverse events in the evidence base were generally of mild to moderate severity, differences in incidence could potentially have important implications for treatment tolerability and adherence in clinical practice. For instance, reduced adherence due to adverse effects may, in turn, influence the long-term effectiveness of migraine prophylaxis and indirectly impact broader health outcomes. Among the most common adverse events encountered with topiramate use in patients with migraine are decreased appetite and paresthesia (tingling or numbness) [34, 35]. Valproate may show side effects of weight gain, dizziness and tremors [36, 37]. Increased appetite with low-dose valproate therapy is noted predominantly in young patients [36]. The teratogenic effect of valproate strictly limits its use for pregnant patients with migraine [37, 38]. Valproate use necessitates careful avoidance in women of childbearing potential, not only due to the risk of harm to the fetus, but also in light of the hypercoagulable state of pregnancy, as valproate may increase vascular risk in migraineurs. The use of topiramate has also been limited for prophylaxis of migraine during pregnancy due to growing evidence of malformations, autism spectrum disorder and intellectual disability resulting from its use [39]. Taken together, these adverse-event profiles highlight the importance of individualised treatment planning, especially for women of reproductive age.

Beyond tolerability, the metabolic and vascular implications of these therapies may also influence treatment selection. Migraine, particularly migraine with aura, has been consistently associated with an increased risk of ischaemic stroke, highlighting the importance of considering vascular risk when selecting prophylactic therapies. In this context, the metabolic effects of valproate and topiramate may have clinically relevant implications beyond migraine control. Valproate has been associated with weight gain, hyperinsulinaemia, and the development of polycystic ovary syndrome, all of which may contribute to an adverse cardiometabolic profile and potentially increase long-term vascular risk. In contrast, topiramate is frequently associated with weight loss and may confer favourable effects on metabolic parameters. However, its use has also been linked to metabolic acidosis and kidney stones. These differing profiles suggest that treatment selection should take into account individual patient characteristics, particularly in those with pre-existing vascular risk factors such as obesity, hypertension, or diabetes.

Our findings are consistent with recent reviews on migraine prevention, including studies comparing topiramate and valproate. Cui et al., concluded that there was no significant difference between the effectiveness of topiramate and valproate [33]. A review by Lampl et al. comparing various migraine prevention therapies found both topiramate and valproate to be effective, with some studies suggesting a slight edge for newer options, such as monoclonal antibodies targeting calcitonin gene-related peptide or its receptor [15]. Similarly, a review by Robblee et al., indicated that monoclonal antibodies targeting CGRP pathways outperform older drugs like topiramate and divalproex in reducing monthly migraine days [40]. Whilst these emerging therapies were not included in our systematic review, the findings from these newer treatments provide a perspective on the evolving landscape of migraine prophylaxis. They suggest that while topiramate and valproate are effective, they may not offer the same level of benefit as the latest therapies.

### Strengths

The strengths of this systematic review lie in its comprehensive search strategy and inclusion of studies across multiple countries and languages, providing insights into the global applicability of valproate and topiramate for patients with migraine. The inclusion of both English and non-English studies adds depth to the findings. Additionally, the review rigorously assessed the risk of bias across all studies, providing transparency about the quality of the evidence.

### Limitations

This review has several limitations. Notably, a meta-analysis was not conducted and the available evidence was judged to carry a substantial risk of bias. As a result, the conclusions drawn from this review should be interpreted with caution. In addition, a high degree of clinical, methodologic and statistical heterogeneity was noted. There was also a lack of change-from-baseline data in many trials, which makes direct comparisons difficult, given that baseline imbalances were also seen. Furthermore, short and inconsistent definitions of follow-ups complicate the interpretation of long-term efficacy and safety profiles. The trials also relied heavily on self-reported measures, which introduced potential bias, as many were not adequately blinded or were open-label. High dropout rates and small sample sizes were additional methodological concerns. Additionally, the populations included in the studies predominantly consisted of female participants, limiting the generalisability of the findings to male migraineurs. Sex-stratified descriptions or analyses were also not possible due to a lack of reporting by the included trials, which further limits the finding’s interpretability regarding male migraineurs. We also lacked access to individual participant data (IPD). The use of IPD may have led to more meaningful comparisons. Moreover, the absence of quantitative synthesis limits the ability to compare effect sizes across studies and may reduce the precision of the findings for clinical decision-making. Although this approach was chosen to avoid potentially misleading pooled estimates in the presence of heterogeneity and bias, alternative approaches, such as descriptive or statistical syntheses, may have provided additional quantitative context. An additional limitation relates to the external validity and clinical contextualisation of the included evidence. The included trials were conducted in a limited set of countries, which may restrict the generalisability of findings to broader populations outside of these regions. Potential differences in genetic polymorphisms affecting drug metabolism, as well as dietary patterns and lifestyle factors that influence cardiometabolic and vascular risk, could likely modify treatment effects in broader populations. This necessitates the need for rigorous trials across more regions to further explore the efficacy and safety profiles of these drugs. Furthermore, baseline vascular risk factors, such as blood pressure, lipid profiles, glycaemic status, and smoking history, were either inconsistently reported or not reported at all across trials, and no study evaluated how these parameters changed during treatment. This limits the ability to contextualise findings within a cerebrovascular risk framework and assess long-term adverse event profiles. In addition, the key migraine outcomes assessed were predominantly patient-reported and therefore susceptible to reporting and recall bias, particularly in the absence of adequate blinding. None of the included studies incorporated objective measures, which could have provided a more detailed insight into the treatment effects. As a result, the broader cerebrovascular implications of these interventions remain uncertain. Also, although the scope of this review was restricted to adults-only to maintain sufficient clinical homogeneity, relevance and internal validity (and also because valproate is currently only FDA-approved (FDA: Food and Drug Administration) for adult migraine and not pediatric migraine), this means that data pertaining to the pediatric population was not assessed in this review and, as such, findings from this study should not be extrapolated to individuals less than 18 years of age [41–43]. Lastly, the very low certainty of evidence for all outcomes could mean that the true effects may favour either intervention, or show no significant differences.

### Future directions

Several avenues for future research can be identified based on the limitations and gaps in the current review. Future trials should prioritise well-controlled designs with prospective registrations, rigorous randomisation, blinding, and detailed reporting that is in line with contemporary reporting guidelines such as the Consolidated Standards of Reporting Trials (CONSORT) statement. Change-from-baseline data should be provided to account for baseline imbalances, per-protocol analyses should be avoided, and standardised patient-centered core outcome sets should be considered. Future trials should also have larger numbers of participants, informed by transparent a priori sample size calculations that incorporate anticipated effect sizes and clinically meaningful differences. Conducting more multicentric studies would further enhance population representativeness, minimise centre-specific bias, and strengthen the external validity of the evidence base. Studies assessing the long-term effectiveness and safety of valproate and topiramate are also needed, as many of the studies in the review had relatively short follow-up periods. Additionally, the role of combination therapies, including valproate and topiramate, needs investigation.

## Conclusion

Both valproate and topiramate appear effective in reducing migraine frequency, duration, pain intensity, and associated disability. However, the available head-to-head evidence is insufficient to establish the superiority of either drug, and interpretation is limited by substantial heterogeneity and methodological weaknesses across studies. Thus, in the absence of definitive comparative efficacy, clinical decision-making may be better guided by differences in safety profiles, drug availability, and individual patient characteristics and preferences. Given the global burden of migraine, further high-quality, adequately powered comparative trials reporting standardised, core-outcome sets are needed to better inform optimal prophylactic treatment strategies.

## Supplementary Information

Supplementary Material 1.

## Data Availability

No new datasets have been currently generated for this study.

## Abbreviations

BMI: Body mass index
CI: Confidence interval
CONSORT: Consolidated Standards of Reporting Trials
FDA: Food and Drug Administration
HIT-6: Headache Impact Test-6
ICHD-3: International Classification of Headache Disorders, 3rd Edition
IHS: International Headache Society
IPD: Individual participant data
MIDAS: Migraine Disability Assessment
MD: Mean difference
NRS: Numeric Rating Scale
OSF: Open Science Framework
PRISMA: Preferred Reporting Items for Systematic Reviews and Meta-Analyses
RCT: Randomized controlled trial
RoB 2: Revised Cochrane Risk of Bias tool (version 2)
RR: Risk ratio
SD: Standard deviation
VAS: Visual analog scale
WHO ICTRP: World Health Organization International Clinical Trials Registry Platform
