# Supplementary material for: Prophylactic efficacy and safety of valproate versus topiramate for migraine: a systematic review
Source: BMC Neurol. 2026 May 20;26:571. doi: 10.1186/s12883-026-04990-7 (PMC13556092; doi:10.1186/s12883-026-04990-7)
Supplement: Supplementary file 1 [file 12883_2026_4990_MOESM1_ESM.docx]

**Supplementary Materials**

**Supplementary Table S1.** PRISMA Checklist

| **Section and Topic** | **Item #** | **Checklist item** | **Location where item is reported** |
| --- | --- | --- | --- |
| **TITLE** | | |  |
| Title | 1 | Identify the report as a systematic review. | Title |
| **ABSTRACT** | | |  |
| Abstract | 2 | See the PRISMA 2020 for Abstracts checklist. | See Below |
| **INTRODUCTION** | | |  |
| Rationale | 3 | Describe the rationale for the review in the context of existing knowledge. | Page 2 |
| Objectives | 4 | Provide an explicit statement of the objective(s) or question(s) the review addresses. | Page 2 |
| **METHODS** | | |  |
| Eligibility criteria | 5 | Specify the inclusion and exclusion criteria for the review and how studies were grouped for the syntheses. | Page 3 |
| Information sources | 6 | Specify all databases, registers, websites, organisations, reference lists and other sources searched or consulted to identify studies. Specify the date when each source was last searched or consulted. | Page 3 |
| Search strategy | 7 | Present the full search strategies for all databases, registers and websites, including any filters and limits used. | Supplementary |
| Selection process | 8 | Specify the methods used to decide whether a study met the inclusion criteria of the review, including how many reviewers screened each record and each report retrieved, whether they worked independently, and if applicable, details of automation tools used in the process. | Page 3 |
| Data collection process | 9 | Specify the methods used to collect data from reports, including how many reviewers collected data from each report, whether they worked independently, any processes for obtaining or confirming data from study investigators, and if applicable, details of automation tools used in the process. | Page 3 |
| Data items | 10a | List and define all outcomes for which data were sought. Specify whether all results that were compatible with each outcome domain in each study were sought (e.g. for all measures, time points, analyses), and if not, the methods used to decide which results to collect. | Page 4 |
|  | 10b | List and define all other variables for which data were sought (e.g. participant and intervention characteristics, funding sources). Describe any assumptions made about any missing or unclear information. | Page 4 |
| Study risk of bias assessment | 11 | Specify the methods used to assess risk of bias in the included studies, including details of the tool(s) used, how many reviewers assessed each study and whether they worked independently, and if applicable, details of automation tools used in the process. | Page 4 |
| Effect measures | 12 | Specify for each outcome the effect measure(s) (e.g. risk ratio, mean difference) used in the synthesis or presentation of results. | Page 4 |
| Synthesis methods | 13a | Describe the processes used to decide which studies were eligible for each synthesis (e.g. tabulating the study intervention characteristics and comparing against the planned groups for each synthesis (item #5)). | Page 4 |
|  | 13b | Describe any methods required to prepare the data for presentation or synthesis, such as handling of missing summary statistics, or data conversions. | Page 4 |
|  | 13c | Describe any methods used to tabulate or visually display results of individual studies and syntheses. | Page 4 |
|  | 13d | Describe any methods used to synthesize results and provide a rationale for the choice(s). If meta-analysis was performed, describe the model(s), method(s) to identify the presence and extent of statistical heterogeneity, and software package(s) used. | Page 4 |
|  | 13e | Describe any methods used to explore possible causes of heterogeneity among study results (e.g. subgroup analysis, meta-regression). | Page 4 |
|  | 13f | Describe any sensitivity analyses conducted to assess robustness of the synthesized results. | Page 4 |
| Reporting bias assessment | 14 | Describe any methods used to assess risk of bias due to missing results in a synthesis (arising from reporting biases). | Risk of bias figures given in supplementary |
| Certainty assessment | 15 | Describe any methods used to assess certainty (or confidence) in the body of evidence for an outcome. | Page 4 |
| **RESULTS** | | |  |
| Study selection | 16a | Describe the results of the search and selection process, from the number of records identified in the search to the number of studies included in the review, ideally using a flow diagram. | Figure 1 |
|  | 16b | Cite studies that might appear to meet the inclusion criteria, but which were excluded, and explain why they were excluded. | Figure 1 |
| Study characteristics | 17 | Cite each included study and present its characteristics. | Table 1 |
| Risk of bias in studies | 18 | Present assessments of risk of bias for each included study. | Supplementary |
| Results of individual studies | 19 | For all outcomes, present, for each study: (a) summary statistics for each group (where appropriate) and (b) an effect estimate and its precision (e.g. confidence/credible interval), ideally using structured tables or plots. | Supplementary |
| Results of syntheses | 20a | For each synthesis, briefly summarise the characteristics and risk of bias among contributing studies. | Page 6 |
|  | 20b | Present results of all statistical syntheses conducted. If meta-analysis was done, present for each the summary estimate and its precision (e.g. confidence/credible interval) and measures of statistical heterogeneity. If comparing groups, describe the direction of the effect. | Page 7 |
|  | 20c | Present results of all investigations of possible causes of heterogeneity among study results. | Page 14 |
|  | 20d | Present results of all sensitivity analyses conducted to assess the robustness of the synthesized results. | Systematic review |
| Reporting biases | 21 | Present assessments of risk of bias due to missing results (arising from reporting biases) for each synthesis assessed. | Risk of bias figures given in supplementary |
| Certainty of evidence | 22 | Present assessments of certainty (or confidence) in the body of evidence for each outcome assessed. | Page 14 |
| **DISCUSSION** | | |  |
| Discussion | 23a | Provide a general interpretation of the results in the context of other evidence. | Page 14 |
|  | 23b | Discuss any limitations of the evidence included in the review. | Page 15 |
|  | 23c | Discuss any limitations of the review processes used. | Page 15 |
|  | 23d | Discuss implications of the results for practice, policy, and future research. | Pages 16 |
| **OTHER INFORMATION** | | |  |
| Registration and protocol | 24a | Provide registration information for the review, including register name and registration number, or state that the review was not registered. | Page 3 |
|  | 24b | Indicate where the review protocol can be accessed, or state that a protocol was not prepared. | Page 3 |
|  | 24c | Describe and explain any amendments to information provided at registration or in the protocol. | Page 5 |
| Support | 25 | Describe sources of financial or non-financial support for the review, and the role of the funders or sponsors in the review. | Title page |
| Competing interests | 26 | Declare any competing interests of review authors. | Title page |
| Availability of data, code and other materials | 27 | Report which of the following are publicly available and where they can be found: template data collection forms; data extracted from included studies; data used for all analyses; analytic code; any other materials used in the review. | Title page |
| **PRISMA 2020 for Abstracts Checklist** | | | |
| **TITLE** |  |  | **Reported (Yes/No)** |
| Title | 1 | Identify the report as a systematic review. | Yes |
| **BACKGROUND** |  |  |  |
| Objectives | 2 | Provide an explicit statement of the main objective(s) or question(s) the review addresses. | Yes |
| **METHODS** |  |  |  |
| Eligibility criteria | 3 | Specify the inclusion and exclusion criteria for the review. | Yes |
| Information sources | 4 | Specify the information sources (e.g. databases, registers) used to identify studies and the date when each was last searched. | Yes |
| Risk of bias | 5 | Specify the methods used to assess risk of bias in the included studies. | Yes |
| Synthesis of results | 6 | Specify the methods used to present and synthesise results. | Yes |
| **RESULTS** |  |  |  |
| Included studies | 7 | Give the total number of included studies and participants and summarise relevant characteristics of studies. | Yes |
| Synthesis of results | 8 | Present results for main outcomes, preferably indicating the number of included studies and participants for each. If meta-analysis was done, report the summary estimate and confidence/credible interval. If comparing groups, indicate the direction of the effect (i.e. which group is favoured). | Yes |
| **DISCUSSION** |  |  |  |
| Limitations of evidence | 9 | Provide a brief summary of the limitations of the evidence included in the review (e.g. study risk of bias, inconsistency and imprecision). | Yes |
| Interpretation | 10 | Provide a general interpretation of the results and important implications. | Yes |
| **OTHER** |  |  |  |
| Funding | 11 | Specify the primary source of funding for the review. | Yes |
| Registration | 12 | Provide the register name and registration number. | Yes |

**Supplementary Table S2.** Source(s) with Search Strings and Results

| **Database(s)** | **Search String** | **Results** |
| --- | --- | --- |
| ***PubMed*** | #1  ((((((((((((((((((("Topiramate"[Mesh]) OR (Topiramat*[Title/Abstract])) OR (Epitomax[Title/Abstract])) OR (USL255[Title/Abstract])) OR ("USL-255"[Title/Abstract])) OR (Topamax[Title/Abstract])) OR ("McN-4853"[Title/Abstract])) OR (McN4853[Title/Abstract])) OR (Topiratore[Title/Abstract])) OR (Qudexy*[Title/Abstract])) OR (Trokendi*[Title/Abstract])) OR (Eprontia[Title/Abstract])) OR ("RWJ-17021*"[Title/Abstract])) OR (RWJ17021*[Title/Abstract])) OR ("ET-101"[Title/Abstract])) OR (ET101[Title/Abstract])) OR ("SPN-538"[Title/Abstract])) OR (SPN538[Title/Abstract])) OR ("KW-6485"[Title/Abstract])) OR (KW6485[Title/Abstract])  #2  (((((((((((((((((((((((("Valproic Acid"[Mesh]) OR (Valpro*[Title/Abstract])) OR ("2-Propylpentan*"[Title/Abstract])) OR ("Di-n-propylacet*"[Title/Abstract])) OR ("Dipropyl Acetate"[Title/Abstract])) OR (DipropylAcetate[Title/Abstract])) OR (Divalproex[Title/Abstract])) OR (Depakote[Title/Abstract])) OR (Vupral[Title/Abstract])) OR (Ergenyl[Title/Abstract])) OR (Depakene[Title/Abstract])) OR (Convulsofin[Title/Abstract])) OR (Depakine[Title/Abstract])) OR (Propylisopropylacet*[Title/Abstract])) OR (Absenor[Title/Abstract])) OR (apilepsin[Title/Abstract])) OR (Avugane[Title/Abstract])) OR (Valparin[Title/Abstract])) OR (Epilim[Title/Abstract])) OR (Convulex[Title/Abstract])) OR (Deprakine[Title/Abstract])) OR (Encorate[Title/Abstract])) OR (Stavzor[Title/Abstract])) OR (Orfiril[Title/Abstract])) OR (Valparin[Title/Abstract])  #3  ((((((("Migraine Disorders"[Mesh]) OR (Migrain*[Title/Abstract])) OR ("Status Migrainosus"[Title/Abstract])) OR ("Sick Headache"[Title/Abstract])) OR ("Sick Headaches"[Title/Abstract])) OR (Migralep*[Title/Abstract])) OR ("migrainous headache*"[Title/Abstract])) OR ("status hemicranicus"[Title/Abstract])  #4  (((((("Randomized Controlled Trial" [Publication Type]) OR ("Randomized Controlled Trials as Topic"[Mesh])) OR (random*)) OR (RCT)) OR (RCTs)) OR ("RC-trial")) OR ("RC-trials")  #5  #1 AND #2 AND #3 AND #4 | **63** |
| ***Embase*** | ('migraine'/exp OR 'familial migraine' OR 'headache, migrainous' OR 'hemicrania' OR 'migraine' OR 'migraine disorders' OR 'migrainous headache' OR 'status hemicranicus') AND ('topiramate'/exp OR '(4, 4, 11, 11 tetramethyl 3, 5, 7, 10, 12 pentaoxatricyclo [7.3.0.0 (2, 6)] dodecan 6 yl) methyl sulfamate' OR '2, 3:4, 5 bis o (1 methylethylidene) beta dextro fructopyranose sulfamate' OR '2, 3:4, 5 di o isopropylidene beta dextro fructopyranose sulfamate' OR 'acomicil' OR 'ecuram' OR 'epiramat' OR 'epitomax' OR 'epitoram' OR 'eprontia' OR 'erravia' OR 'et 101' OR 'et101' OR 'etopro' OR 'fagodol' OR 'jadix' OR 'kw 6485' OR 'kw6485' OR 'lusitrax' OR 'maritop' OR 'mcn 4853' OR 'mcn4853' OR 'oritop' OR 'piraleps' OR 'pirantal' OR 'pirepil' OR 'qudexy' OR 'qudexy xr' OR 'ramas (drug)' OR 'rwj 17021' OR 'rwj 17021-000' OR 'rwj17021' OR 'rwj17021-000' OR 'sincronil' OR 'spn 538' OR 'spn538' OR 'talopam' OR 'tiramat' OR 'topaben' OR 'topamac' OR 'topamax' OR 'topamax sprinkle' OR 'topepsil' OR 'topibrain' OR 'topilek' OR 'topimark' OR 'topimax' OR 'topina' OR 'topiramat' OR 'topiramat-ct' OR 'topiramate' OR 'topiramato' OR 'topiratore' OR 'topit' OR 'toramat' OR 'torlepta' OR 'trokendi' OR 'trokendi xr' OR 'trokesa' OR 'usl 255' OR 'usl255') AND ('valproic acid'/exp OR '2 propylpentanoate' OR '2 propylpentanoic acid' OR '2 propylvalerate sodium' OR '2 propylvaleric acid' OR '2 propylvaleric acid sodium' OR '2, 2 dipropyl acetic acid' OR '4 heptanecarboxylic acid' OR 'abbott 44090' OR 'absenor' OR 'absenor depot' OR 'alpha propylvalerate' OR 'alpha propylvaleric acid' OR 'apilepsin' OR 'avugane' OR 'baceca' OR 'convulex' OR 'cs 1' OR 'cs1' OR 'ct 010' OR 'ct010' OR 'delepsine' OR 'depacon' OR 'depakene' OR 'depakin' OR 'depakine' OR 'depakine druppels' OR 'depakine enteric' OR 'depakine iv' OR 'depalept' OR 'deprakine' OR 'deproic' OR 'di n propylacetate' OR 'di n propylacetate sodium' OR 'di n propylacetic acid' OR 'diplexil' OR 'dipropyl acetic acid' OR 'dipropylacetate' OR 'dipropylacetate sodium' OR 'dipropylacetatic acid' OR 'dipropylacetic acid' OR 'diprosin' OR 'dyzantil' OR 'encorate' OR 'epiject' OR 'epilam' OR 'epilex' OR 'epilim' OR 'epilim enteric' OR 'epilim liquid' OR 'episenta' OR 'ergenyl' OR 'espa valept' OR 'espa-valept' OR 'everiden' OR 'goilim' OR 'hexaquin' OR 'k 828' OR 'k 828ab' OR 'k 828sp' OR 'k828' OR 'k828ab' OR 'k828sp' OR 'kw 6066 n' OR 'labazene' OR 'leptilan' OR 'leptilanil' OR 'micropakine' OR 'mylproin' OR 'myproic acid' OR 'n dipropylacetic acid' OR 'npl 2005' OR 'npl2005' OR 'orfil' OR 'orfiril' OR 'orfiril iv' OR 'orlept' OR 'petilin' OR 'propylisopropylacetic acid' OR 'propymal' OR 'propymal enteric' OR 'savicol' OR 'sodium 2 propylpentanoate' OR 'sodium 2 propylvalerate' OR 'sodium di n propyl acetate' OR 'sodium di n propylacetate' OR 'sodium dipropyl acetate' OR 'sodium dipropylacetate' OR 'sodium n dipropylacetate' OR 'sodium valproate' OR 'stavzor' OR 'syonell' OR 'val 001 (anticonvulsive agent)' OR 'val001 (anticonvulsive agent)' OR 'valcote' OR 'valepil' OR 'valeptol' OR 'valerin' OR 'valoin' OR 'valpakine' OR 'valparin' OR 'valporal' OR 'valprax' OR 'valpro' OR 'valpro al' OR 'valpro beta' OR 'valproat' OR 'valproat ct' OR 'valproat-ct' OR 'valproate' OR 'valproate sodium' OR 'valprodura' OR 'valproic acid' OR 'valprosid' OR 'valprotek' OR 'valsup' OR 'vupral') AND ('randomized controlled trial'/exp OR 'controlled trial, randomized' OR 'randomised controlled study' OR 'randomised controlled trial' OR 'randomized controlled study' OR 'randomized controlled trial' OR 'trial, randomized controlled' OR 'rct' OR 'rcts' OR 'random*') | **354** |
| ***Scopus*** | #1  TITLE-ABS-KEY(Topiramate OR Topiramat* OR Epitomax OR USL255 OR "USL-255" OR Topamax OR "McN-4853" OR McN4853 OR Topiratore OR Qudexy* OR Trokendi* OR Eprontia OR "RWJ-17021*" OR RWJ17021* OR "ET-101" OR ET101 OR "SPN-538" OR SPN538 OR "KW-6485" OR KW6485)  #2  TITLE-ABS-KEY(Valpro* OR "2-Propylpentan*" OR "Di-n-propylacet*" OR "Dipropyl Acetate" OR DipropylAcetate OR Divalproex OR Depakote OR Vupral OR Ergenyl OR Depakene OR Convulsofin OR Depakine OR Propylisopropylacet* OR Absenor OR Apilepsin OR Avugane OR Valparin OR Epilim OR Convulex OR Deprakine OR Encorate OR Stavzor OR Orfiril)  #3  TITLE-ABS-KEY(Migrain* OR "Status Migrainosus" OR "Sick Headache" OR "Sick Headaches" OR Migralep* OR "migrainous headache*" OR "status hemicranicus")  #4  TITLE-ABS-KEY("randomized controlled trial" OR "randomised controlled trial" OR random* OR RCT OR RCTs OR "RC-trial" OR "RC-trials")  #5  #1 AND #2 AND #3 AND #4 | **332** |
| ***Web of Science*** | #1  TS=(Topiramate OR Topiramat* OR Epitomax OR USL255 OR "USL-255" OR Topamax OR "McN-4853" OR McN4853 OR Topiratore OR Qudexy* OR Trokendi* OR Eprontia OR "RWJ-17021*" OR RWJ17021* OR "ET-101" OR ET101 OR "SPN-538" OR SPN538 OR "KW-6485" OR KW6485)  #2  TS=(Valpro* OR "2-Propylpentan*" OR "Di-n-propylacet*" OR "Dipropyl Acetate" OR DipropylAcetate OR Divalproex OR Depakote OR Vupral OR Ergenyl OR Depakene OR Convulsofin OR Depakine OR Propylisopropylacet* OR Absenor OR Apilepsin OR Avugane OR Valparin OR Epilim OR Convulex OR Deprakine OR Encorate OR Stavzor OR Orfiril)  #3  TS=(Migrain* OR "Status Migrainosus" OR "Sick Headache" OR "Sick Headaches" OR Migralep* OR "migrainous headache*" OR "status hemicranicus")  #4  TS=("randomized controlled trial" OR "randomised controlled trial" OR random* OR RCT OR RCTs OR "RC-trial" OR "RC-trials")  #5  #1 AND #2 AND #3 AND #4 | **112** |
| ***Cochrane*** | ID Search Hits  #1 MeSH descriptor: [Topiramate] explode all trees 691  #2 (Topiramate OR Topiramat* OR Epitomax OR USL255 OR "USL-255" OR Topamax OR "McN-4853" OR McN4853 OR Topiratore OR Qudexy* OR Trokendi* OR Eprontia OR RWJ NEXT 17021* OR RWJ17021* OR "ET-101" OR ET101 OR "SPN-538" OR SPN538 OR "KW-6485" OR KW6485):ti,ab,kw 1700  #3 #1 OR #2 1700  #4 MeSH descriptor: [Valproic Acid] explode all trees 1157  #5 (Valpro* OR 2 NEXT Propylpentan* OR Di NEXT n NEXT propylacet* OR "Dipropyl Acetate" OR DipropylAcetate OR Divalproex OR Depakote OR Vupral OR Ergenyl OR Depakene OR Convulsofin OR Depakine OR Propylisopropylacet* OR Absenor OR Apilepsin OR Avugane OR Valparin OR Epilim OR Convulex OR Deprakine OR Encorate OR Stavzor OR Orfiril):ti,ab,kw 3080  #6 #4 OR #5 3080  #7 MeSH descriptor: [Migraine Disorders] explode all trees 3944  #8 (Migrain* OR "Status Migrainosus" OR "Sick Headache" OR "Sick Headaches" OR Migralep* OR migrainous NEXT headache* OR "status hemicranicus"):ti,ab,kw 10493  #9 #7 OR #8 10493  #10 MeSH descriptor: [Random Allocation] explode all trees 25208  #11 MeSH descriptor: [Randomized Controlled Trial] explode all trees 34  #12 MeSH descriptor: [Randomized Controlled Trials as Topic] explode all trees 61652  #13 ("randomized controlled trial" OR "randomised controlled trial" OR random* OR RCT OR RCTs OR "RC-trial" OR "RC-trials"):ti,ab,kw 1425068  #14 #10 OR #11 OR #12 OR #13 1425192  #15 #3 AND #6 AND #9 AND #14 47 | **47** |
| ***ClinicalTrials.gov*** | (Topiramate OR Topiramat OR Epitomax OR USL255 OR USL-255 OR Topamax OR McN-4853 OR McN4853 OR Topiratore OR Qudexy OR Trokendi OR Eprontia OR RWJ-17021 OR RWJ17021 OR ET-101 OR ET101 OR SPN-538 OR SPN538 OR KW-6485 OR KW6485) AND (Valproate OR 2-Propylpentanoic acid OR Di-n-propylacetate OR Dipropyl-Acetate OR DipropylAcetate OR Divalproex OR Depakote OR Vupral OR Ergenyl OR Depakene OR Convulsofin OR Depakine OR Propylisopropylacete OR Absenor OR Apilepsin OR Avugane OR Valparin OR Epilim OR Convulex OR Deprakine OR Encorate OR Stavzor OR Orfiril)  This search string was entered in the ‘Expert Search’ search field. | **30** |
| ***WHO ICTRP*** | (valproate OR valproic) AND topiramate AND migraine | **5** |
| ***Websites*** | Topiramate AND (Valproate OR Valproic)  This Search String Was Adapted To These Websites*:  scholar.google.com, researchgate.net, academia.edu, SID.ir, oversea.cnki.net, qikan.cqvip.com, search.bvsalud.org  Where appropriate, supplemental searches in Persian, Chinese and Spanish were also conducted. These terms were used (the words were selected and searched in consultation with native speakers having medical backgrounds):   1. Persian: والپروات, توپیرامات 2. Chinese: 丙戊酸/丙戊酸, 托吡酯/托吡酯 3. Spanish: valproico/valproato, topiramato | **23** |
| ***Citation Chaining*** | Done Through Google Scholar | **6** |

**Supplementary Figures SA1-SA14.** Risk-of-bias Assessments: ‘Traffic Light’ Plots and ‘Summary’ Plots

**Migraine Frequency**

**Supplementary Figures SA1-SA2.** Risk-of-bias Assessments: ‘Traffic Light’ Plots and ‘Summary’ Plots

**Migraine Duration**

**Supplementary Figures SA3-SA4.** Risk-of-bias Assessments: ‘Traffic Light’ Plots and ‘Summary’ Plots

**Migraine Pain Intensity**

**Supplementary Figures SA5-SA6.** Risk-of-bias Assessments: ‘Traffic Light’ Plots and ‘Summary’ Plots

**Migraine HIT6 Score**

**Supplementary Figures SA7-SA8.** Risk-of-bias Assessments: ‘Traffic Light’ Plots and ‘Summary’ Plots

**Migraine MIDAS Score**

**Supplementary Figures SA9-SA10.** Risk-of-bias Assessments: ‘Traffic Light’ Plots and ‘Summary’ Plots

**Responder Rate**

**Supplementary Figures SA11-SA12.** Risk-of-bias Assessments: ‘Traffic Light’ Plots and ‘Summary’ Plots

**Adverse Events**

**Supplementary Figures SA13-SA14.** Risk-of-bias Assessments: ‘Traffic Light’ Plots and ‘Summary’ Plots

| **Study Characteristics** | | **Continuous Outcomes** | | | | | | | | | | | | | | | | | | | | | | | | | | | | | | | | | | | | |
| --- | --- | --- | --- | --- | --- | --- | --- | --- | --- | --- | --- | --- | --- | --- | --- | --- | --- | --- | --- | --- | --- | --- | --- | --- | --- | --- | --- | --- | --- | --- | --- | --- | --- | --- | --- | --- | --- | --- |
|  |  | **Study Arm** | **Migraine Frequency** | | | | | | | **Migraine Duration** | | | | | | | **Migraine Pain Intensity** | | | | | | | | **Migraine HIT-6 Score** | | | | | | | **Migraine MIDAS Score** | | | | | | |
| **Author** | **Year** |  | **Baseline** | | | **Last Timepoint** | | |  | **Baseline** | | | **Last Timepoint** | | |  | **Baseline** | | | **Scale** | **Last Timepoint** | | |  | **Baseline** | | | **Last Timepoint** | | |  | **Baseline** | | | **Last Timepoint** | | |  |
|  |  |  | **Mean** | **SD** | **Total** | **Mean** | **SD** | **Total** |  | **Mean** | **SD** | **Total** | **Mean** | **SD** | **Total** |  | **Mean** | **SD** | **Total** |  | **Mean** | **SD** | **Total** |  | **Mean** | **SD** | **Total** | **Mean** | **SD** | **Total** |  | **Mean** | **SD** | **Total** | **Mean** | **SD** | **Total** |  |
| Afshari et al. | 2012 | Valproate | 7.5 | 1.9 | 28 | 3.6 | 1.8 | 28 |  | 13.5 hours | 13.7 | 28 | 7.5 hours | 4.1 | 28 |  | 8.6 | 1.7 | 28 | VAS | 6.3 | 1.9 | 28 |  | 65.8 | 5 | 28 | 57.2 | 6.9 | 28 |  | 18.6 | 15 | 28 | 11.5 | 10.4 | 28 |  |
|  |  |  |  |  |  |  |  |  |  |  |  |  |  |  |  |  |  |  |  |  |  |  |  |  |  |  |  |  |  |  |  |  |  |  |  |  |  |  |
|  |  | Topiramate | 6.8 | 2 | 28 | 3 | 1.9 | 28 |  | 13 hours | 10.9 | 28 | 6 hours | 2.9 | 28 |  | 8.6 | 1.7 | 28 |  | 5.2 | 1.5 | 28 |  | 64.5 | 4.7 | 28 | 49.2 | 8.1 | 28 |  | 18.2 | 13.3 | 28 | 7.6 | 7.8 | 28 |  |
|  |  |  |  |  |  |  |  |  |  |  |  |  |  |  |  |  |  |  |  |  |  |  |  |  |  |  |  |  |  |  |  |  |  |  |  |  |  |  |
| Bartolini et al. | 2005 | Valproate | 27 | 1.3 | 22 | 3.2 | 2.9 | 22 |  | N/R | N/R | N/R | N/R | N/R | N/R |  | N/R | N/R | N/R | N/R | N/R | N/R | N/R |  | N/R | N/R | N/R | N/R | N/R | N/R |  | 25.2 | 3.6 | 22 | 5.7 | 6.4 | 22 |  |
|  |  |  |  |  |  |  |  |  |  |  |  |  |  |  |  |  |  |  |  |  |  |  |  |  |  |  |  |  |  |  |  |  |  |  |  |  |  |  |
|  |  | Topiramate | 26.1 | 2.3 | 22 | 2.76 | 3.9 | 22 |  | N/R | N/R | N/R | N/R | N/R | N/R |  | N/R | N/R | N/R | N/R | N/R | N/R | N/R |  | N/R | N/R | N/R | N/R | N/R | N/R |  | 27.8 | 12.1 | 22 | 7.1 | 10.3 | 22 |  |
|  |  |  |  |  |  |  |  |  |  |  |  |  |  |  |  |  |  |  |  |  |  |  |  |  |  |  |  |  |  |  |  |  |  |  |  |  |  |  |
| Bavrasad et al. | 2010 | Valproate | 10.14 | 1.98 | 35 | 4.81 | 1.7 | 35 |  | 22.13 hours | 6.68 | 35 | 7.27 hours | 6.47 | 35 |  | 9.2 | 1.36 | 35 | NRS | 4.15 | 0.864 | 35 |  | N/R | N/R | N/R | N/R | N/R | N/R |  | Regarding MIDAS score, the study reported that participants receiving topiramate showed greater improvement in performance compared with those receiving sodium valproate, though exact data were not clearly reported in a before-after fashion. | | | | | |  |
|  |  | Topiramate | 10.07 | 2.32 | 35 | 4.58 | 1.1 | 35 |  | 24.37 hours | 7.65 | 35 | 6.23 hours | 5.22 | 35 |  | 9.3 | 1.45 | 35 |  | 4.7 | 1.24 | 35 |  | N/R | N/R | N/R | N/R | N/R | N/R |  |  |  |  |  |  |  |  |
| Choudhary et al. | 2017 | Valproate | 3.64 | 1.19 | 45 | 1.73 | 0.61 | 45 |  | N/R | N/R | N/R | N/R | N/R | N/R |  | N/R | N/R | N/R | N/R | N/R | N/R | N/R |  | N/R | N/R | N/R | N/R | N/R | N/R |  | N/R | N/R | N/R | N/R | N/R | N/R |  |
|  |  |  |  |  |  |  |  |  |  |  |  |  |  |  |  |  |  |  |  |  |  |  |  |  |  |  |  |  |  |  |  |  |  |  |  |  |  |  |
|  |  | Topiramate | 3.55 | 1.22 | 45 | 1.31 | 0.46 | 45 |  | N/R | N/R | N/R | N/R | N/R | N/R |  | N/R | N/R | N/R | N/R | N/R | N/R | N/R |  | N/R | N/R | N/R | N/R | N/R | N/R |  | N/R | N/R | N/R | N/R | N/R | N/R |  |
|  |  |  |  |  |  |  |  |  |  |  |  |  |  |  |  |  |  |  |  |  |  |  |  |  |  |  |  |  |  |  |  |  |  |  |  |  |  |  |
| Fazlalizadeh et al. | 2009 | Valproate | N/R | N/R | N/R | N/R | N/R | N/R |  | N/R | N/R | N/R | N/R | N/R | N/R |  | N/R | N/R | N/R | N/R | N/R | N/R | N/R |  | N/R | N/R | N/R | N/R | N/R | N/R |  | N/R | N/R | N/R | N/R | N/R | N/R |  |
|  |  |  |  |  |  |  |  |  |  |  |  |  |  |  |  |  |  |  |  |  |  |  |  |  |  |  |  |  |  |  |  |  |  |  |  |  |  |  |
|  |  | Topiramate | N/R | N/R | N/R | N/R | N/R | N/R |  | N/R | N/R | N/R | N/R | N/R | N/R |  | N/R | N/R | N/R | N/R | N/R | N/R | N/R |  | N/R | N/R | N/R | N/R | N/R | N/R |  | N/R | N/R | N/R | N/R | N/R | N/R |  |
|  |  |  |  |  |  |  |  |  |  |  |  |  |  |  |  |  |  |  |  |  |  |  |  |  |  |  |  |  |  |  |  |  |  |  |  |  |  |  |
| Ma et al. | 2009 | Valproate | 7.5 | 1.5 | 68 | 2.1 | 1.1 | 68 |  | 14.5 hours | 7.6 | 68 | 6.6 hours | 3.9 | 68 |  | N/R | N/R | N/R | N/R | N/R | N/R | N/R |  | N/R | N/R | N/R | N/R | N/R | N/R |  | N/R | N/R | N/R | N/R | N/R | N/R |  |
|  |  |  |  |  |  |  |  |  |  |  |  |  |  |  |  |  |  |  |  |  |  |  |  |  |  |  |  |  |  |  |  |  |  |  |  |  |  |  |
|  |  | Topiramate | 6.9 | 1.8 | 60 | 2.8 | 1.5 | 60 |  | 15.7 hours | 6.3 | 60 | 9.5 hours | 4.8 | 60 |  | N/R | N/R | N/R | N/R | N/R | N/R | N/R |  | N/R | N/R | N/R | N/R | N/R | N/R |  | N/R | N/R | N/R | N/R | N/R | N/R |  |
|  |  |  |  |  |  |  |  |  |  |  |  |  |  |  |  |  |  |  |  |  |  |  |  |  |  |  |  |  |  |  |  |  |  |  |  |  |  |  |
| Zeinhom et al. | 2025 | Valproate | 9.67 | 2.23 | 300 | 6.67 | 2.98 | 300 |  | 8 hours | 2.98 | 300 | 6.27 hours | 2.09 | 300 |  | 7.33 | 0.74 | 300 | VAS | 5.67 | 1.49 | 300 |  | 59 | 5.96 | 300 | 55.83 | 5.96 | 300 |  | N/R | N/R | N/R | N/R | N/R | N/R |  |
|  |  |  |  |  |  |  |  |  |  |  |  |  |  |  |  |  |  |  |  |  |  |  |  |  |  |  |  |  |  |  |  |  |  |  |  |  |  |  |
|  |  | Topiramate | 9.67 | 2.23 | 300 | 6.33 | 2.23 | 300 |  | 8 hours | 2.98 | 300 | 6.33 hours | 2.23 | 300 |  | 7.33 | 0.74 | 300 |  | 5.33 | 0.74 | 300 |  | 59.67 | 7.45 | 300 | 54.5 | 7.45 | 300 |  | N/R | N/R | N/R | N/R | N/R | N/R |  |
|  |  |  |  |  |  |  |  |  |  |  |  |  |  |  |  |  |  |  |  |  |  |  |  |  |  |  |  |  |  |  |  |  |  |  |  |  |  |  |
| Zhang et al. | 2018 | Valproate | 4.9 | 1.1 | 51 | 3.8 | 0.7 | 51 |  | 8.6 days | 2.4 | 51 | 4.6 days | 1.6 | 51 |  | 7.8 | 2.2 | 51 | VAS | 5.2 | 1.4 | 51 |  | N/R | N/R | N/R | N/R | N/R | N/R |  | N/R | N/R | N/R | N/R | N/R | N/R |  |
|  |  |  |  |  |  |  |  |  |  |  |  |  |  |  |  |  |  |  |  |  |  |  |  |  |  |  |  |  |  |  |  |  |  |  |  |  |  |  |
|  |  | Topiramate | 5.2 | 0.8 | 51 | 2.3 | 0.4 | 51 |  | 8.8 days | 2.3 | 51 | 3.8 days | 1.1 | 51 |  | 7.9 | 2 | 51 |  | 3.5 | 1 | 51 |  | N/R | N/R | N/R | N/R | N/R | N/R |  | N/R | N/R | N/R | N/R | N/R | N/R |  |
|  |  |  |  |  |  |  |  |  |  |  |  |  |  |  |  |  |  |  |  |  |  |  |  |  |  |  |  |  |  |  |  |  |  |  |  |  |  |  |

NRS: Numeric Rating Scale

VAS: Visual Analog Scale

N/R: Not Reported

**Supplementary Table S3:** Table for continuous outcomes, showing baseline values and values reported at last timepoint

| **Author** | **Year** | **Migraine Frequency** | **Migraine Duration** | **Migraine Pain Score** | **Migraine HIT-6 Score** | **Migraine MIDAS Score** |
| --- | --- | --- | --- | --- | --- | --- |
| **Afshari et al.** | **2012** | Over the 12-week follow-up period (from beginning the treatment, follow-up every four weeks as 3 visits), both topiramate and sodium valproate significantly reduced migraine attack frequency. At baseline, patients in the topiramate group experienced 6.8 ± 2.0 attacks per 4 weeks, which decreased to 4.8 ± 2.0 at 4 weeks, 3.4 ± 1.6 at 8 weeks, and 3.0 ± 1.9 at 12 weeks. In the valproate group, frequency declined from 7.5 ± 1.9 at baseline to 4.3 ± 1.9, 3.7 ± 1.8, and 3.6 ± 1.8 at corresponding follow-ups. Between-group differences were not statistically significant across all timepoints (p = 0.163, 0.395, 0.477, 0.252 at baseline and the three visits). However, data were not reported as change from baseline. | At the end of the 12-week follow-up, the average duration per migraine episode had decreased considerably in both treatment arms. For topiramate, mean duration fell from 13.0 ± 10.9 hours at baseline to 10.5 ± 8.2 hours at 4 weeks, 7.9 ± 5.7 hours at 8 weeks, and 6.0 ± 2.9 hours by week 12. In the valproate group, duration declined from 13.5 ± 13.7 hours to 10.9 ± 9.0, 9.2 ± 6.6, and 7.5 ± 4.1 hours, respectively. Although both groups showed clear improvement, the between-group comparison revealed no significant difference at any timepoint (p = 0.877, 0.874, 0.446, 0.15), indicating comparable effectiveness. However, data were not reported as change from baseline. | The Visual Analogue Scale (VAS) was used. Headache intensity decreased progressively over the 12-week follow-up in both groups. In the topiramate group, mean pain scores improved from 8.6 ± 1.7 at baseline to 6.7 ± 2.0 at 4 weeks, 6.2 ± 1.9 at 8 weeks, and 5.2 ± 1.5 at week 12 (p < 0.001 within group). Similarly, in the valproate group, scores declined from 8.6 ± 1.7 to 6.7 ± 1.5, 6.4 ± 1.6, and 6.3 ± 1.9 (p < 0.001 within group). By the final 12-week assessment, the reduction in VAS score was significantly greater for topiramate than for valproate (p = 0.027), demonstrating superior efficacy of topiramate in decreasing headache pain intensity at that time-point. Importantly, however, non-significant differences were seen at other timepoints (p = 1, 0.93, 0.67 respectively). However, data were not reported as change from baseline. | HIT-6 score improved significantly from baseline to end timepoint of measurement in both treatment groups. In the topiramate group, HIT-6 scores decreased from 64.5 ± 4.7 at baseline to 49.2 ± 8.1 at study end, while in the valproate group, scores declined from 65.8 ± 5.0 to 57.2 ± 6.9. Both reductions were statistically significant within groups, but higher with topiramate (p = 0.00). However, this outcome was not pre-specified in the trial registry and data at baseline and week 8 is given, and not at other timepoints. | MIDAS scores improved in both groups by the end timepoint of measurement. In the topiramate arm, the MIDAS score declined from 18.2 ± 13.3 at baseline to 7.6 ± 7.8, while in the valproate group it decreased from 18.6 ± 15.0 to 11.5 ± 10.4. Both groups showed statistically significant within-group reductions, though the between-group difference was not statistically significant (p = 0.128). However, this outcome was not pre-specified in the trial registry and data at baseline and week 8 is given, and not at other timepoints. |
| **Bartolini et al.** | **2005** | At baseline (T0), patients with chronic migraine experienced a high frequency of headache days: 26.1 ± 2.3 days per month in the topiramate group and 27.0 ± 1.3 days per month in the valproate group. After reaching full target doses, significant improvement was observed at both follow-up points. At 1 month (T1) from administration of full-dose, the mean number of headache days per 30-day period was reduced to 10.4 ± 9.3 with topiramate and 6.1 ± 4.5 with valproate, showing better efficacy for valproate (P < 0.02). At 3 months (T2), continued improvement occurred in both groups, reaching 2.76 ± 3.9 days/month for topiramate and 3.2 ± 2.9 days/month for valproate. The overall within-group reductions from baseline were highly significant (P < 0.00001), but no statistically significant difference remained between the two drugs at the 3-month follow-up. However, change from baseline data were not provided. | N/R | N/R | N/R | At baseline (T0), the mean MIDAS score was 27.8 ± 12.1 for the topiramate group and 25.2 ± 3.6 for the valproate group, indicating comparable levels of functional impairment. At the 3-month follow-up (T2) from administration of full-dose, MIDAS scores dropped significantly to 7.1 ± 10.3 with topiramate and 5.7 ± 6.4 with valproate (P < 0.00001 within each group). No significant difference between the two treatments was detected in the final intergroup comparison, showing that both topiramate and valproate markedly improved migraine-related disability after 3 months of therapy. However, change from baseline data were not provided. |
| **Bavrasad et al.** | **2010** | Over the 12-week follow-up period, both topiramate and sodium valproate produced significant reductions in mean monthly migraine frequency. In the topiramate group, the number of headache days per month decreased from 10.07 ± 2.32 at baseline to 4.58 ± 1.11 after treatment (p = 0.013). Similarly, in the valproate group, headache days declined from 10.14 ± 1.98 to 4.81 ± 1.70 (p = 0.028). Both treatments achieved more than 50% reduction in headache frequency for a majority of patients, though the difference between groups was not statistically significant (p = 0.359). However, data were not reported as change from baseline. | At the 12-week follow-up, the average duration of individual migraine attacks was markedly reduced in both treatment groups. In patients receiving topiramate, mean headache duration fell from 24.37 ± 7.65 hours at baseline to 6.23 ± 5.22 hours post-treatment (p = 0.035). The valproate group showed a comparable reduction from 22.13 ± 6.68 hours to 7.27 ± 6.47 hours (p = 0.027). Although the reduction appeared slightly greater in the topiramate group, the between-group difference was not statistically significant (p = 0.524). However, data were not reported as change from baseline. | Pain intensity, rated on a 0 to 10 numeric rating scale (NRS), also improved over the 12-week period. In the topiramate arm, the mean pain score dropped from 9.30 ± 1.45 before treatment to 4.70 ± 1.24 after treatment (p = 0.024). In the valproate group, scores decreased from 9.20 ± 1.36 to 4.15 ± 0.864 (p = 0.018). The decline in headache severity was statistically significant within each group but not significantly different between the two (p = 0.259). However, data were not reported as change from baseline. | N/R | MIDAS scores improved significantly by the 12-week endpoint in both treatment groups. The baseline MIDAS score averaged 53.23 ± 6.52 in the topiramate group and 46.76 ± 4.29 in the valproate group. Baseline scores showed statistically significant difference (p < 0.05). The degree of MIDAS improvement was greater in the topiramate group, but neither the post-treatment values nor change from baseline values were given. |
| **Choudhary et al.** | **2017** | At baseline, the mean number of migraine episodes per month was 3.64 ± 1.19 in the sodium valproate group (Group A) and 3.55 ± 1.22 in the topiramate group (Group B). After six weeks of treatment, the frequency of migraine episodes decreased significantly in both groups: to 1.73 ± 0.61 in the sodium valproate group and 1.31 ± 0.46 in the topiramate group. Although a p-value showing the between-group differences was not shown for this outcome, the authors did conclude that sodium valproate was significantly better in reducing migraine frequency. | N/R | N/R | N/R | N/R |
| **Fazlalizadeh et al.** | **2009** | N/R | N/R | N/R | N/R | N/R |
| **Ma et al.** | **2009** | Before treatment, patients in the sodium valproate group experienced an average of 7.5 ± 1.5 migraine attacks per month, while those in the topiramate group averaged 6.9 ± 1.8 attacks per month. After five months of continuous prophylactic treatment, both groups showed significant reductions in attack frequency. The sodium valproate group’s frequency decreased dramatically to 2.1 ± 1.1 attacks per month, while the topiramate group improved to 2.8 ± 1.5 attacks per month. At the 5-month follow-up, the reduction in monthly migraine frequency was greater in the sodium valproate group (5.4 ± 1.2 attacks/month) compared with the topiramate group (4.1 ± 1.1 attacks/month), and this difference was statistically significant (P < 0.05). Thus, sodium valproate demonstrated superior efficacy in reducing migraine frequency over a 5-month course of treatment. | At baseline, the mean duration of migraine attacks was 14.5 ± 7.6 hours per episode in the sodium valproate group and 15.7 ± 6.3 hours per episode in the topiramate group. After five months of prophylactic therapy, duration of attacks shortened to 6.6 ± 3.9 hours in the sodium valproate group and 9.5 ± 4.8 hours in the topiramate group. When comparing the mean reduction in headache duration between baseline and the 5-month follow-up, sodium valproate again performed better, showing a 7.9 ± 3.3 hour decrease versus 6.2 ± 2.8 hours with topiramate (P < 0.05). These findings suggest that sodium valproate achieved more rapid and sustained improvement in reducing attack duration. | N/R | N/R | N/R |
| **Zeinhom et al.*** | **2025** | At baseline, mean headache frequency was comparable between groups (valproate 9.67 ± 2.23 vs topiramate 9.67 ± 2.23 per month; P = 0.12). After 1 month of treatment, frequency decreased to 7.67 ± 2.23 and 7.67 ± 2.98 per month, respectively (P = 0.29). Further reduction was seen at 2 months (valproate 7.33 ± 2.23; topiramate 7.67 ± 2.98; P = 0.46) and 3 months (valproate 6.67 ± 2.98; topiramate 6.33 ± 2.23; P = 0.74). Within-group improvement was evident over time, but no statistically significant between-group difference was detected at any follow-up. However, data were not reported as change from baseline. | At baseline, mean headache duration was 8.0 ± 2.98 h in both groups (P = 0.22). After 1 month, duration decreased to 7.0 ± 3.72 h with valproate and 7.0 ± 3.72 h with topiramate (P = 0.21). At 2 months, duration was 6.67 ± 2.98 h (valproate) versus 6.93 ± 3.58 h (topiramate) (P = 0.68). By 3 months, duration further declined to 6.27 ± 2.09 h and 6.33 ± 2.23 h, respectively (P = 0.67). Within-group reductions were consistent but without significant intergroup differences across time points. However, data were not reported as change from baseline. | Baseline mean VAS pain scores were identical (7.33 ± 0.74 for both groups). Improvement was observed at 1 month (6.33 ± 0.74 valproate vs 6.0 ± 1.49 topiramate) (P = 0.03), 2 months (5.67 ± 1.49 vs 5.67 ± 1.49; P = 0.01), and 3 months (5.67 ± 1.49 vs 5.33 ± 0.74; P = 0.004), showing significantly greater pain reduction with topiramate. However, data were not reported as change from baseline. | Mean HIT-6 scores were similar at baseline (59 ± 5.96 valproate vs 59.67 ± 7.45 topiramate; P = 0.87). After 1 month, scores decreased to 56.67 ± 7.45 and 56.27 ± 7.3 (P = 0.12). Continued improvement occurred at 2 months (56 ± 5.96 vs 54.67 ± 7.45) (P = 0.01) and 3 months (55.83 ± 5.96 vs 54.5 ± 7.45) (P = 0.004), with topiramate producing significantly greater improvement from the second month onward. However, data were not reported as change from baseline. | N/R |
| **Zhang et al.** | **2018** | At baseline, the average monthly migraine frequency was 4.9 ± 1.1 attacks per month in the sodium valproate group and 5.2 ± 0.8 attacks per month in the topiramate group. After three months of prophylactic treatment, attack frequency decreased in both groups. Patients receiving sodium valproate experienced a reduction to 3.8 ± 0.7 attacks per month, while those receiving topiramate improved more markedly to 2.3 ± 0.4 attacks per month. The between-group difference at the 3-month follow-up was statistically significant (P < 0.05), indicating that topiramate provided a greater reduction in migraine frequency than sodium valproate. However, the study did not calculate values as change from baseline, though the baseline imbalances were negligible. | Before treatment, the mean duration of each migraine attack was 8.6 ± 2.4 days in the sodium valproate group and 8.8 ± 2.3 days in the topiramate group. Following three months of continuous therapy, attack duration was shortened in both treatment arms. The sodium valproate group showed a decrease to 4.6 ± 1.6 days, whereas the topiramate group exhibited a greater improvement, with attack duration reduced to 3.8 ± 1.1 days. At the 3-month follow-up, the difference between the two treatments was statistically significant (P < 0.05), again favoring topiramate. However, the study did not calculate values as change from baseline, though the baseline imbalances were negligible. | Pain intensity, measured on a visual analog scale (VAS), averaged 7.8 ± 2.2 in the sodium valproate group and 7.9 ± 2.0 in the topiramate group at baseline. After three months of treatment, the VAS score decreased to 5.2 ± 1.4 for the sodium valproate group and to 3.5 ± 1.0 for the topiramate group. Analysis in the study found a more substantial reduction in perceived pain intensity with topiramate, with differences at the 3-month follow-up reaching statistical significance (P < 0.05). However, the study did not calculate values as change from baseline, though the baseline imbalances were negligible. | N/R | N/R |

* The study reported continuous outcomes as medians with interquartile ranges. Using meta-accelerator, they were converted to means with standard deviations.

N/R = Not reported

**Supplementary Table S4:** Table for continuous outcomes, summarizing results at each reported timepoint

**Supplementary Table S5:** Table for responder rate

| **Study Label** | | **Group** | **Responder Rate** |
| --- | --- | --- | --- |
| **Author** | **Year** |  |  |
| Afshari et al. | 2012 | Valproate | 18 out of 28 participants were responders. |
|  |  | Topiramate | 20 out of 28 participants were responders. The difference was not statistically significant (P > 0.05). |
| Bartolini et al. | 2005 | Valproate | 21 out of 22 participants were responders. |
|  |  | Topiramate | 21 out of 22 participants were responders. |
| Bavrasad et al. | 2010 | Valproate | No significant difference was found between the responder rates among the two groups (P > 0.05). |
|  |  | Topiramate |  |
| Choudhary et al. | 2017 | Valproate | 29 out of 45 participants were responders. |
|  |  | Topiramate | 16 out of 45 participants were responders. P-value was 0.006, indicating a statistically significant difference. |
| Fazlalizadeh et al. | 2009 | Valproate | 262 out of 285 patients (91.9%) in the sodium valproate group were responders. However, patients who had previously failed two or more therapeutic regimens were not included in the trial population and this could explain the unusually high responder rates. |
|  |  | Topiramate | 266 out of 284 patients (93.7%) in the topiramate group were responders. However, patients who had previously failed two or more therapeutic regimens were not included in the trial population and this could explain the unusually high responder rates. Additionally, the different was reported as not being statistically significant (P > 0.05). |
| Ma et al. | 2009 | Valproate | 45 out of 68 patients (66.2%) were responders. A statistically significant difference was reported in the study, with P-value being mentioned as < 0.05 for this outcome. |
|  |  | Topiramate | 32 out of 60 patients (53.3%) were responders. |
| Zeinhom et al. | 2025 | Valproate | 67 out of 300 (22.3%) were responders. |
|  |  | Topiramate | 82 out of 300 (27.3%) were responders. |
| Zhang et al. | 2018 | Valproate | N/R |
|  |  | Topiramate | N/R |

**Supplementary Table S6:** Table for adverse events

| **Study Label** | | **Group** | **Adverse Events** |
| --- | --- | --- | --- |
| **Author** | **Year** |  |  |
| Afshari et al. | 2012 | Valproate | 78.6% (22/28) of participants who completed the study reported at least one adverse event. The most frequently observed events were increased appetite (46.4%), hair loss (32.1%), somnolence (25%), nausea (21.4%), and vertigo (21.4%). Other commonly reported effects included paresthesia, memory disturbance, tremor, and eye pain. Less frequent events were anxiety, dry mouth, and decreased visual acuity. Rare events included dysarthria and diarrhea, while no cases of ataxia were observed. In addition, from the 8 patients from the valproate group who dropped out of the study, 6 patients reported experiencing adverse events when contacted by phone for the reason of their withdrawal. |
|  |  | Topiramate | 64.3% (18/28) of participants who completed the study reported at least one adverse event. The most frequently observed events were decreased appetite (42.9%), paresthesia (35.7%), and vertigo (25%). Other commonly reported effects included fatigue, somnolence, and nausea. Less frequent events were memory disturbance, tremor, eye pain, and dry mouth. Rare events included anxiety and ataxia, and decreased visual acuity. No cases of diarrhea were observed. In addition, from the 12 patients from the topiramate group who dropped out of the study, 2 patients stated that they experienced adverse events. |
| Bartolini et al. | 2005 | Valproate | 3 patients from the valproate group dropped out due to feeling drowsiness. The trial reported that the drug was well-tolerated. |
|  |  | Topiramate | 2 patients from the topiramate group dropped out due to feeling drowsiness, and 1 patient dropped out due to gastric discomfort and paresthesias. The trial reported that the drug was well-tolerated. |
| Bavrasad et al. | 2010 | Valproate | In the sodium valproate group, the most frequently observed adverse events included drowsiness (4.28%), nausea (5.71%), tremor (4.28%), weight gain (2.85%), and hair loss (2.85%). One participant in the valproate group dropped out due to severe drowsiness and nausea associated with valproate use. |
|  |  | Topiramate | Among participants receiving topiramate, the most commonly reported medical complications were paresthesia (11.4%), drowsiness (11.4%), weight loss (2.9%), and dizziness (4.3%). One participant was withdrawn from the study due to severe paresthesia attributed to topiramate. |
| Choudhary et al. | 2017 | Valproate | N/R |
|  |  | Topiramate | N/R |
| Fazlalizadeh et al. | 2009 | Valproate | Adverse drug reactions occurred in 40 patients (14%) in the sodium valproate group. 19 people experienced general side effects, 14 experienced hepatic side effects, and 7 experienced dermatologic side effects. No immunologic side effects were reported. |
|  |  | Topiramate | Adverse drug reactions occurred in 41 patients (14.4%) in the topiramate group. 29 people experienced general side effects, 6 experienced hepatic side effects, and 6 experienced immunologic side effects. No dermatologic side effects were reported. |
| Ma et al. | 2009 | Valproate | 6 out of 68 patients (8.8%) experienced adverse events: Bradycardia (3 cases), hypotension (2 cases), dizziness (1 case). |
|  |  | Topiramate | 14 out of 60 patients (23.3%) experienced at least one adverse event. Limb paresthesia (6 cases), loss of appetite (5 cases), somnolence (2 cases), dizziness (3 cases). |
| Zeinhom et al. | 2025 | Valproate | In the valproate arm, 129/300 participants (43.0%) were patients with any adverse effects. The most frequently reported effects included increased appetite (15.3%), mood abnormalities (3.7%), menstrual irregularities (3.3%), hair loss (2.7%), vomiting (2.3%), and elevated liver enzymes (2.3%). Less common events were tremor, gastritis, diarrhoea, anxiety, and numbness. Rare events such as renal stones and lethargy were also noted. Notably, 23/300 participants (7.7%) discontinued the treatment due to adverse effects. |
|  |  | Topiramate | In the topiramate arm, 66/300 participants (22.0%) had any adverse events (which was significantly less than the valproate arm). The most frequent events were decreased appetite (4.3%), numbness (3.0%), memory impairment (1.8%), sleep disturbance (1.7%), and vomiting (1.7%). Less commonly observed events included diarrhoea, tremor, eye pain, and renal stones. Rare events such as hair loss, elevated liver enzymes, and lethargy were reported infrequently. |
| Zhang et al. | 2018 | Valproate | Total incidence: 10/51 (19.6%). Fatigue (3 cases). Dizziness (3 cases). Loss of appetite (4 cases). |
|  |  | Topiramate | Total incidence: 8/51 (15.7%) in topiramate group. Fatigue (3 cases). Dizziness (2 cases). Loss of appetite (3 cases). |

**Supplementary Table S7.** Summary of Findings (SoF) Table.

**Summary of findings:** Valproate versus Topiramate for migraine prophylaxis in adult migraineurs

**Population:** Adults with migraine

**Study Design:** Randomized controlled trials (RCTs)

**Intervention:** Valproate

**Comparison:** Topiramate

| **Outcomes** | **Anticipated absolute effects (95% CI)** | **Relative effect (95% CI)** | **Number of studies** | **Certainty of the evidence (GRADE)** | **Comments** |
| --- | --- | --- | --- | --- | --- |
| **Migraine frequency** | Not pooled. | Not pooled. | 7 studies | **Very low**^a,b^ | There was mixed evidence regarding comparative efficacy. Both interventions reduced migraine frequency, with no consistent difference between groups. |
| **Responder rate** | Not pooled. | Not pooled. | 7 studies | **Very low**^a,b^ | Responder rates were clinically meaningful in both groups, but direction of effect varied widely across studies. |
| **Migraine duration** | Not pooled. | Not pooled. | 5 studies | **Very low**^a,b^ | Both treatments reduced migraine duration. No consistent superiority was observed. |
| **Pain intensity** | Not pooled. | Not pooled. | 4 studies | **Very low**^a,b^ | There was a tendency towards greater reduction in pain intensity with topiramate in some studies, though findings were not consistent. |
| **HIT-6** | Not pooled. | Not pooled. | 2 studies | **Very low**^a,c^ | Both drugs improved disability scores. Results were inconsistent and limited. |
| **MIDAS** | Not pooled. | Not pooled. | 3 studies | **Very low**^a,c^ | Both drugs improved disability scores. Results were inconsistent and limited. |
| **Adverse events** | Not pooled. | Not pooled. | 7 studies | **Very low**^a,b^ | Overall incidence of adverse events appeared comparable, though the types of adverse events differed between drugs. |

**GRADE Working Group grades of evidence**

**High certainty:** we are very confident that the true effect lies close to that of the estimate of the effect.

**Moderate certainty:** we are moderately confident in the effect estimate; the true effect is likely to be close to the estimate of the effect, but there is a possibility that it is substantially different.

**Low certainty:** our confidence in the effect estimate is limited; the true effect may be substantially different from the estimate of the effect.

**Very low certainty:** we have very little confidence in the effect estimate; the true effect is likely to be substantially different from the estimate of effect.

**Reasons for Downgrading**

^a^ Downgraded twice due to high risk of bias.

^b^ Downgraded due to inconsistency.

^c^ Downgraded due to imprecision.
